# Potential for Layered Double Hydroxides-Based, Innovative Drug Delivery Systems

**DOI:** 10.3390/ijms15057409

**Published:** 2014-04-29

**Authors:** Kai Zhang, Zhi Ping Xu, Ji Lu, Zhi Yong Tang, Hui Jun Zhao, David A. Good, Ming Qian Wei

**Affiliations:** 1 School of Medical Science & Griffith Health Institute, Gold Coast Campus, Griffith University, Southport, QLD 4222, Australia; E-Mail: kai.zhang4@griffithuni.edu.au; 2 Australian Institutes for Bioengineering & Nanotechnology, University of Queensland, St Lucia, QLD 4072, Australia; E-Mails: gordonxu@uq.edu.au (Z.P.X.); ji.lu@uqconnect.edu.au (J.L.); 3 National Centre for Nanoscience and Technology, Chinese Academy of Sciences, Beijing 100190, China; E-Mail: zytang@nanoctr.cn; 4 Griffith Schools of Environment, Gold Coast Campus, Griffith University, Southport, QLD 4222, Australia; E-Mail: h.zhao@griffith.edu.au; 5 School of Physiotherapy, Faculty of Health Science, Australian Catholic University, Brisbane, QLD 4014, Australia

**Keywords:** layered double hydroxides (LDHs), drug delivery, gene therapy, chemotherapy

## Abstract

Layered Double Hydroxides (LDHs)-based drug delivery systems have, for many years, shown great promises for the delivery of chemical therapeutics and bioactive molecules to mammalian cells *in vitro* and *in vivo.* This system offers high efficiency and drug loading density, as well as excellent protection of loaded molecules from undesired degradation. Toxicological studies have also found LDHs to be biocompatible compared with other widely used nanoparticles, such as iron oxide, silica, and single-walled carbon nanotubes. A plethora of bio-molecules have been reported to either attach to the surface of or intercalate into LDH materials through co-precipitation or anion-exchange reaction, including amino acid and peptides, ATPs, vitamins, and even polysaccharides. Recently, LDHs have been used for gene delivery of small molecular nucleic acids, such as antisense, oligonucleotides, PCR fragments, siRNA molecules or sheared genomic DNA. These nano-medicines have been applied to target cells or organs in gene therapeutic approaches. This review summarizes current progress of the development of LDHs nanoparticle drug carriers for nucleotides, anti-inflammatory, anti-cancer drugs and recent LDH application in medical research. Ground breaking studies will be highlighted and an outlook of the possible future progress proposed. It is hoped that the layered inorganic material will open up new frontier of research, leading to new nano-drugs in clinical applications.

## 1. Introduction

Layered Double Hydroxides (LDHs), also known as hydrotalcite-like (HTI), hydrotalcite-type (HTt) or anionic clays belong to a big family of layered materials [1]. The first member of this natural mineral family was identified in Sweden in 1842, which is known as hydrotalcite with the formula Mg_6_Al_2_(OH)_16_CO_3_·4H_2_O [2,3]. Most of the LDH materials can be described as a general formula [M^II^_1−_*_x_*M^III^*_x_*(OH)_2_*^x^*^+^(A*^m^*^−^)*_x_*_/_*_m_*·*n*H_2_O] (*x* = 0.2–0.4; *n* = 0.5–1), where M^II^ represents a divalent metal cation, M^III^ a trivalent metal cation and A*^m^*^−^ an anion [4,5]. Structurally, like brucite Mg(OH)_2_, each cation in LDH layers is surrounded with six OH^−^ ions forming a octahedral subunit, and every two subunits share edges and could expand the two-dimensional layer, theoretically, to infinity [6]. Anions A*^m^*^−^ located between two layers balance the positive charge of cations via electrostatic interaction. With the contribution of hydrogen bonds between layers, the two layers are held together forming a three-dimensional structure.

Interestingly, not only can the layer cations of these materials be replaced among a wide range selection of cations, but also the anions located at the interlayer are believed to have possible substitutions among organic or inorganic, simple or complex anions, polyoxo materials and simple anionic coordination compounds [7–9]. Furthermore, a unique property, which makes them diverse from other cationic-layered materials, is that they are capable of recovering the double-layered structure after thermal decomposition under mild conditions [10]. All these properties, such as high chemical versatility, anionic exchange capacity, and low cytotoxicity, are leading to a promising future in drug delivery and release, opening up wide possibilities for researches and development for its clinical application. Compared with old-fashioned drug delivery methods which are suffering from problems such as drug degradation, poor bioavailability, and low circulation stability, the LDHs, as a new drug carrier, is much simpler to synthesize in the laboratory, have a high drug transportation efficiency, high drug loading density, low toxicity to target cells or organs and excellent protection to loaded molecules from undesired enzymatic degradation [4]. The earliest application of LDH materials in human therapy was as antiacid and antipeptic reagent [4,11–13]. However, recently LDHs have been employed for clinical disease diagnosis, chemical industry and as a drug carrier responsible for delivering therapeutic and bioactive molecules such as peptides, anti-inflammatory drugs, and even small nucleic acids to mammalian cells *in vitro* or *in vivo* with the purpose of crossing the cell membrane into the cytoplasm [14–16].

## 2. Layered Double Hydroxides in Gene Delivery

### 2.1. Gene Delivery Overview

**Figure 1.**
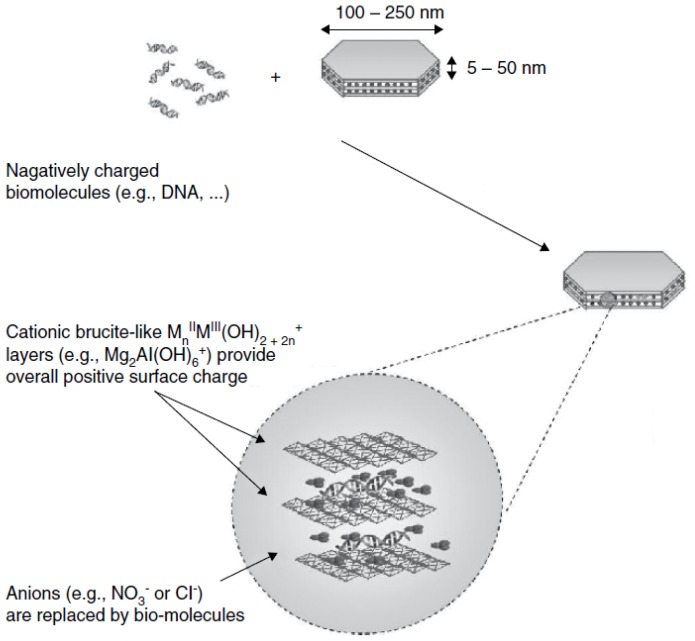
The intercalation mechanism of oligonucleotides with LDH nanoparticles. (Reproduced with permission from Katharina Ladewig *et al.* [4]).

Given that the anions locating at the LDH interlayer can be replaced by other organic or inorganic, simple or complex anions, it is possible that the negatively charged oligonucleotides can be intercalated into the LDH inner space via ion-exchange mechanism (Figure 1). Moreover, it has also been shown that the more guanine and cytosine the small DNA/siRNA possessed, the higher intercalation efficiency achieved [17]. Once the oligonucleotide was intercalated into the LDHs nanoparticles, the host was able to protect the loaded nucleotide from the attack of DNase. On the other hand, there has been an ongoing debate regarding the manner of the association between large nucleotides and the LDH host. In several studies, it was believed that the DNA plasmids were intercalated into LDH layers [18–21]. However, we found that lager DNA fragments (1000 bp or more) or plasmid DNA vectors adapted a secondary structure in an aquatic environment forming a supercoiled structure, which were not completely accessible to intercalation in the interlayer galleries by means of anion exchange [4,22]. Given that observed plasmid/LDH aggregation and lower-than-theoretically possible DNA: LDH ratio, DNA molecules might be more adsorbed to the surface of LDH carriers via ionic bond rather than intercalated into LDH layers. Therefore, the hypothesis of employing LDHs for cellular delivery of large DNA and protection from unexpected degradation might need to be further confirmed.

**Figure 2.**
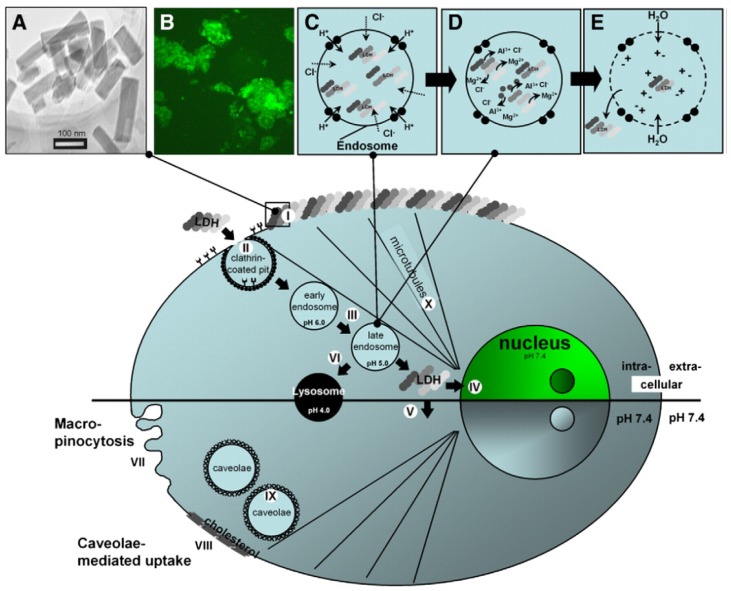
Schematic illustration of LDH-FITC nanoparticle intracellular fate. (**A**) TEM image of LDH-FITC nanoparticles; (**B**) CHO cells transfected with LDH-FITC nanoparticles; (**C**) Pumping protons into the endosome to facilitate acidification for subsequent proteolysis of nutrients, followed by an influx of chloride ions; (**D**) Acid-driven dissolution of LDH-FITC nanoparticles in the late endosome to buffer acidification and ions were released; (**E**) Entrance of water molecules to the endosome due to an increase in ionic strength, leading to osmotic swelling and endosome burst, which releases LDH-FITC nanoparticles into the cytoplasm. Step I. Adhesion of LDH-FITC nanoparticles to the cell membrane; II. Clathrin-mediated endocytosis; III. Endosomal changes; IV. Nuclear localization of LDH-FITC; V. Cytoplasmic distribution of LDH-FITC; VI. Lysosomal pathway; VII. Unspecific uptake through macropinocytosis; VIII–IX. Caveolae-mediated endocytosis; X. Microtubule directing thefreed LDH-FITC nanoparticles to the nucleus. (Cited with permission from Xu *et al.* [23].)

Naked gene delivery has been confronted with hurdles, such as DNA/siRNA instability resulting in unexpected degradation, low cell membrane penetration efficiency, and inability to reach the nuclear zone for gene expression. It is believed that the LDH-mediated cell transfection not only can protect the DNA/siRNA from immature degradation, but also be able to effectively penetrate cell membrane and readily release the nucleotides into cytoplasm near the nucleus, leading to high transfection efficiency and prolonged gene expression. Choy *et al.* firstly revealed the cellular uptake mechanism of the LDH nanoparticles (~150 nm), they were shown to primarily internalize into cells via an energy-dependent endocytic pathway, clathrin-mediated endocytosis [10]. Interestingly, given that the clathrin-mediated endocytosis is the most common internalization pathway in all mammalian cells, the LDH nanoparticle might be able to pass through all type of cells membrane, suggesting its high cell penetration capability as drug delivery system. It is possible that the LDH is transported to endosomes and subsequently to the Golgi apparatus and/or lysosomes after internalization, following the typical clathrin-mediated trafficking pathway, which was illustrated by us (Figure 2) [23]. It has been proved that the gene expression or specific gene silencing occurred six to eight hours after the LDH-mediated cell transfection, suggesting that the LDH host could efficiently release the DNA/siRNA into cytoplasm [20,24]. The rapid gene expression/silencing could be explained by several steps: (1) The DNA/siRNA loaded LDHs hybrids (50~250 nm), which provided overall positive charge, could be attracted by the negatively charged cell membrane and rapidly adhered to the surface of the membrane; (2) After the adsorption, the LDH host could efficiently penetrate the cell membrane via clathrin endocytosis pathway and quickly enter the cytoplasm; (3) Eventually, the DNA/siRNA could be released near the nucleus when the LDH host dissolves, therefore the plasmids could easily to enter the nuclear zone resulting in expression or targeting specific mRNA for gene silencing [23]. The cellular uptake of LDHs increased in a concentration-dependent manner up to the dose of 200 μg/mL. This could be explained by the receptor-mediated endocytosis employed by the LDHs internalization mechanism. Moreover, the cellular uptake amount of LDHs was highly particle size-dependent, the clathrin-mediated endocytosis would select the LDH nanoparticles ranged from 50 to 200 nm for penetrating cells and could achieve high uptake and long retention in cells, whereas the LDHs over 200 nm did not select specific cellular entry [10].

### 2.2. Small Nucleotide Intercalation with LDH Nanoparticles

In 2000, Choy *et al.* firstly intercalated c-myc (a gene overexpressed in cancer cells) antisense oligonucleotide into the inner layers of Mg-Al LDH nanoparticles simply via ion-exchange reaction [18]. LDHs nanoparticle were prepared via co-precipitation of mixing Mg/Al ions with NaOH (pH = 10). After the ion-exchange reaction, an extended basal spacing was obtained indicating that the oligonucleotide was successfully intercalated into LDH layers. Cellular internalization experiments were then performed by employing FITC as a reporter molecule; a significant uptake of cellular DNA/LDH hybrid was observed. Moreover, the longer the time of cell exposure to LDH hybrids, the greater the cellular uptake rate. These experiments went on to prove that, not only could the c-myc/LDH hybrids be internalized by cells, but also the antisense oligonucleotides were involved in cell metabolism. They exposed HL-60 cells to the AS-myc-LDH hybrids at a concentration of 20 μM for four days. A strong suppression of cancer cells growth (65%) was detected, showing that the LDH nanoparticles are able to deliver small nucleic acid into target cells. They also found that such inhibition of cell growth was time and dose-dependent [23].

Small interfering RNAs (siRNAs) have emerged in medical research for diseases caused by single gene mutation because of its ability of silencing a specific gene, yet its application was limited due to their instability both *in vitro* and *in vivo* [25]. In 2010, Ladewig *et al.* employed the Mg/Al-LDH nanoparticles as siRNA carrier in order to penetrate mammalian cells membrane for specific gene silencing [26]. The anti-MAPK1 (ERK2) siRNA/LDH complexes were synthesized following Xu *et al.*’s protocol published in 2006 [27]. HEK293Tcells were transfected with anti-MAPK1 siRNAs using both Lipofectamine and LDH nanoparticles and harvested after 8–48 h incubation. Western blotting showed that a significant ERK2 gene knockdown occurred after eight hours siRNA/LDH exposure and lasted at least another eight hours. After 24 h incubation, the knockdown of target gene expression was still able to be detected in cells transfected with siRNA/Lipofectamine; whereas relatively weak gene suppression were observed in cells exposed to the siRNA/LDH nanoparticles. These results showed that the siRNA/LDH could achieve a faster and more effective target gene knockdown with less functioning time, suggesting that the LDH drug delivery system possessed higher membrane-penetrating and drug controlled-releasing efficiency *in vitro*.

**Figure 3.**
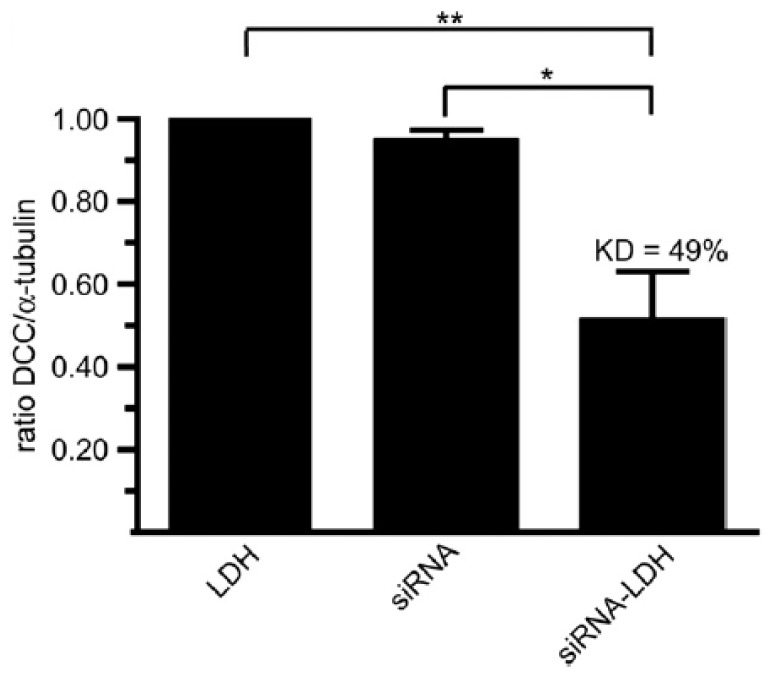
The knockdown profile of DCC by LDH mediated delivery of siRNA to neurons. (Reproduced with permission from Wong *et al.* [24]).

The LDH system was employed to deliver siRNA into cortical neurons in order to develop gene therapies targeting neurological diseases caused by single gene mutation such as Huntington’s disease [24]. The siRNA sequence was covalently coupled to the 6FAM fluorophore at the 5′ end. Then the siRNA/LDH hybrids were synthesized at 37 °C in an aquatic environment and the drug loading was achieved at a mass ratio of 1:1 (siRNA: LDH). Cortical neurons were then exposed to 1.0 μg/mL of siRNA/LDH for 4 and 24 h. We found that over 80% of neurons were transfected with 6FAM-siRNA/LDH complexes in four hours but no significant increase was observed after 24-h incubation. Furthermore, the Deleted in Colorectal Cancer (DCC) gene knockdown was carried out on cortical neurons. When cells were transfected with siRNA/LDH hybrids for further 48 h, an average knockdown efficiency of 49% on cortical neurons was obtained, leading to a conclusion that efficient, targeted gene silencing in neurons can be achieved by LDH mediated siRNA delivery (Figure 3).

### 2.3. Large Nucleotides Intercalation with LDH Nanoparticles

It was also demonstrate that even relatively larger DNA fragments (100–500 bp) could be associated with LDH nanoparticles. Desigaux *et al.* investigated the interaction of DNA fragments with Mg/Al, Mg/Fe and Mg/Ga nanoparticles separately [19]. DNA/LDH hybrids were synthesized as described [18]. LDH/DNA hybrids were recovered after 48 h of aging by ultracentrifugation at 13,000 rpm, extended interlayer distances were detected in all three LDH systems, from ~0.77 nm of all nitrate parent LDHs to ~2.11 nm (Mg/Al), ~1.80 nm (Mg/Fe), and ~1.96 nm (Mg/Ga), respectively. They further explored the Mg/Ga LDH’s ability of delivering DNA fragments into carcinoma HeLa cells. DNA fragments including short fragments, long fragments and DNA plasmid were modified with fluorescent signal YOYO-1 and intercalated into Mg/Ga LDH nanoparticles. Carcinoma HeLa cells were exposed to the fluorescent DNA/LDH complexes for two hours and examined under microscope. Most of fluorescent signals could be clearly observed in the cell cytoplasm and the cell nucleus as well as around the cell membrane. These data directly proved that the relatively larger nucleotides or even DNA plasmids could be efficiently accumulated within cells by employing LDH mediated delivery.

**Figure 4.**
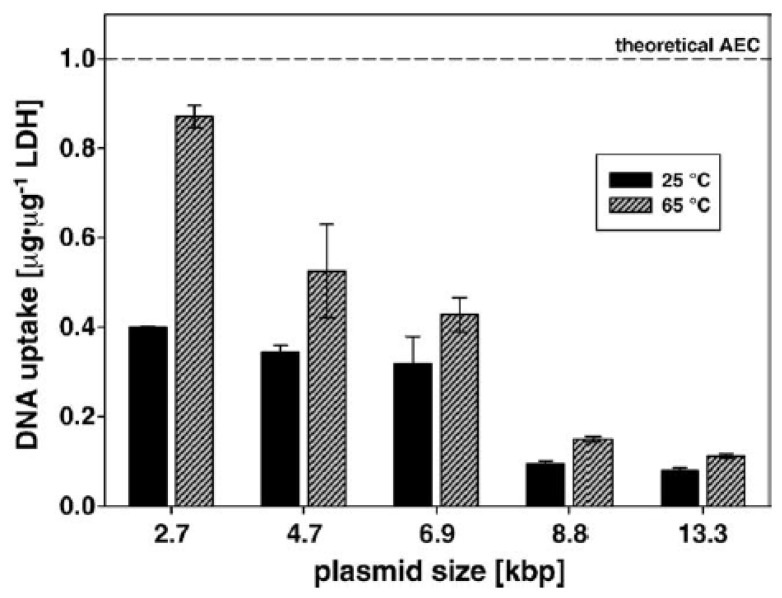
The LDH loading efficiency of LDH with plasmids in various sizes under different experimental conditions. (Reproduced with permission from Xu *et al.* [28])

The interaction of DNA plasmids in various size with Mg_2_Al(OH)_6_NO_3_ LDH nanoparticles under different experimental conditions was investigated by Ladewig *et al.* [28]. With the increasing size of plasmid, less plasmids loading efficiency was evident (Figure 4). Moreover, the variation of experimental condition such as increasing the temperature during the reaction can increase the LDH loading ability, yet an expected enhanced LDH loading capacity (DNA:LDH ratio) was not observed. This could be accounted for by the incompletion of anion change between NO_3_^−^ with DNA even through in some case the amount of DNA is far more than required, which is much different from the case of small DNA fragments [29–32].

Masarudin *et al.* have successfully transfected Vero3 (African monkey kidney) cells with the plasmid pEGFP-N_2_, which contains the GFP gene employing Mg/Al-LDH nanoparticles [20]. The cells were exposed to the plasmid-LDH hybrids (0.1 mg/mL) for 24 h at 37 °C and a clear fluorescence was observed under microscope, indicating that not only was the pEGFP-N_2_ plasmid safely delivered into targeted cells by the LDH carriers, but also could be successfully expressed and involved in cell metabolism. Furthermore, the delivery efficiency of LDH was compared to another commercial transfection reagent, Lipofectamine™ 2000. The Vero3 cells were treated with plasmid-LDH hybrids and Lipofectamine-plasmid mixture containing same amount of plasmid for 48 h. Fluorescence was observed at 6–8 h post-treatment on LDH group, whereas it took 10 h to detect the signal from Lipofectamine group. Even though the transfection efficiency between both vectors was comparably similar, the authors pointed out that, from the cost-operative point of view, their system still benefited. It is indeed a large advantage that the LDH system can be synthesized with low cost reagent and loading drug could be easily achieved via the anion exchange mechanism.

## 3. The Delivery of Non-Steroidal Anti-Inflammatory Drugs (NSAIDs)

Non-steroidal anti-inflammatory drugs are aromatic organic compounds associating with carboxylic groups that can be easily ionized; therefore it is possible that these drugs can be intercalated into LDH layers simply via ion-exchange [33]. It is believed that the synthesis of the NSAIDs/LDH hybrids could improve drug solubility in an aquatic environment and enhance drug absorption by living organisms [34].

### 3.1. The LDH Application with Fenbufen

Fenbufen (FBF) is normally used in relieving cancer pains as well as in the treatment of rheumatoid arthritis and osteoarthritis [35]. However, their negative effect to both the gastrointestinal tract and the central nervous system limit its application. The synthesis of the FBF/LDH complexes with controlled release is expected to alleviate these side effects [36].

In 2004, Li *et al.* first intercalated FBF into the inner layer of the LDH nanoparticles by co-precipitation in a nitrogen environment [37]. Given that the original gallery height is 0.39 nm and the thickness of brucite-like layer itself is 0.48 nm [18,38], XRD analysis showed that the spacing between LDH layers expanded to 1.87 nm after intercalation with FBF, which was consistent with previous study [39]. A significant impact on the FBF-LDH intercalation was observed regarding under differing pH value. The space increased from 1.87 to 3.00 nm while the pH value increased from 8 to 13, suggesting a possible change of interlayer FBF from a monolayer to a bilayer structure. They also investigated the effect of varying the chemical composition of the host layers to the intercalation efficiency of FBF. The same interlayer spacing of 2.35 nm was obtained regardless of different chemicals used to synthesize LDH.

The FBF intercalations with Mg, Al-LDH and Mg, Al, Fe-LDH have been explored by Del Arco *et al.* following three different preparation methods, namely co-precipitation, ion-exchange, and reconstruction, as well as the solubility of the drug/LDH complex in a water environment [40]. In spite of a failed synthesis of FBF/Mg-Al-Fe LDH using reconstruction method (the present of Fe inhibited the intercalation of the drug into LDH); other routes achieved a drug loading range of 31%–44%. In addition, the presence of the LDH was proven to significantly enhance the solubility of FBF in an aquatic environment, irrespective of the LDH acting as an additive or hosting matrix.

The controlled release study of FBF/LDH hybrids was also performed by Li *et al.* [41]. Both Mg/Al-LDH and Li/Al-LDH were employed to intercalate with FBF and were re-suspended in phosphate buffer at pH 7.8. They observed that both hybrids triggered fast drug release in the first 10 min and reached the maximum amount of 40% (Li/AL-LDH) and 59% (Mg/AL-LDH) over 120 min, indicating that the Mg/AL-LDH hybrids release system were more efficient. What is more, the slower linear increase after the rapid release of FBF from the Mg/Al-LDH inferred that the Mg/Al-LDH was a more sustained system for drug release, which suggests that Mg/Al-LDH materials are more suitable for a controlled-release host. However, the application of LDH can be limited in the stomach (pH 1.2). Taking this into account, Evans *et al.* coated the Mg/Al-LDH with enteric polymers and explored the controlled release of the new complexes [41]. The pure FBF/LDH hybrids and the coated complex were separately re-suspended in an aqueous medium with an initial pH at 1.2 for 2 h followed by 6.8 for another 2 h, and eventually at 7.4 for additional 5 h. They found that pure FBF/LDH hybrids achieved a complete FBF release within a very short time, which proved that pure LDH was not capable of delivering drugs in certain realistic conditions; whereas the modified complexes survived at pH 1.2 and gradually released FBF while the pH increased. However, only the FBF-LDH coated with Eudragit S 100 managed to appear in a liner manner and finally peaked at 70% release efficiency over 9 h, suggesting a sustained but slow drug release occurred. It can be concluded that unmodified LDH nanoparticles are not versatile carriers in application, and some materials used for LDH modification somehow reduced the LDH drug release capability. Therefore, finding appropriate LDH modification methods for specific conditions is crucial for their further application.

The profile of LDH nanoparticles loaded with other NSAID, such as Naproxen, Flurbiprofen, Ibuprofen, Diclofenac, Indomethacin, were widely investigated as well [42–48]. In combination of discussion about Fenbufen and Naproxen, there are three possible conclusions: (1) NSAID-LDH synthesis; Two different methods were employed for LDH synthesis, co-precipitation and reconstruction. It is believed that co-precipitation showed more advantages over reconstruction. The co-precipitation lead to a perfect single layered structure whereas the reconstructed LDH could be “contaminated” by other unwanted anions (a layered MgAl-CO_3_ phase existed in MgAl-OH), also the co-precipitation LDHs possessed a larger gallery than the reconstructed LDHs, indicating a relatively higher drug loading efficiency [45,48]; (2) Improvement of loaded drug “survival” ability; it has been discovered that the drug-loaded LDH could remarkably enhance the drug solubility in either aquatic environment [46] or in gastric fluid environment [47]. The thermo-gravimetric analysis also showed that drugs appeared to be more stable after the intercalation with LDH nanoparticles. The presence of LDH nanoparticles could not only prevent loaded drugs from unexpected degradation, but most importantly, lead to an enhancement of gastric mucus permeation and protection of gastrointestinal mucus from ulcer-genic activity [44,46]; (3) Sustained release of drugs; controlled drug release could be achieved in both gastric environment (pH 1~2) and intracellular environment (~pH 7.5). As discussed above, appropriate modification is required for drug release to appear in a liner manner at pH value of 1~2 [41]. However, at a pH value of 7.5, the different mechanism, ion exchange, was the most responsible for the sustained drug release [46].

## 4. The Delivery of Anti-Cancer Drugs

Chemotherapies with cytotoxic drugs have been widely developed and employed for cancer treatment. Yet, the frequent employment of these drugs often leads to the development of cancer cell resistance to these agents during treatment [49–51]. The complex nature of cancer cells presents a great obstacle in developing ideal cancer chemotherapy with high drug efficacy and low side effects [52]. However, it might be achieved by drug-LDH nano-hybrids. The application of LDH nanoparticles for cancer therapy with several commonly used anti-cancer drugs is summarized.

### 4.1. The Delivery of Methotrexate (MTX)

Methotrexate (MTX) is an anti-metabolite and anti-folate drug, which is able to lead abnormal cells through programmed cell death by effectively interfering with cell metabolism. These have been used in treatment for certain human cancers, such as osteosarcoma (bone cancer) and leukemia [53].

Choy *et al.* have explored the intercalation of MTX with LDH nanoparticles in a series of studies [50,54,55]. It has been demonstrated that the cellular uptake of MNNG/HOS (osteosarcoma cells) to LDH-associated MTX was significantly enhanced [56]. The concentration of MTX in MTX-LDH treated cells was considerably higher than that in the cells treated with MTX only at all incubation time. Moreover, cytotoxicity test showed that MTX-LDH was more toxic than MTX only to MNNG/HOS cells [55], as well as Sao-2 and MG-63 cells [54]. These results indicated that the MTX-LDH nano-hybrids could penetrate the cell membrane more effectively than MTX only, leading to enhanced drug efficacy. One more thing to be noted here is that the LDH nanoparticles along had no influence to cell viability at levels up to 500 μg/mL.

Given that the MTX could deactivate the cells metabolism in the manner of inhibiting DNA synthesis and eventually contribute to anti-proliferation [56], these authors also investigated the effect of MTX and MTX-LDH on cell cycle distribution to further confirm the MTX-LDH efficacy [55]. Cells were incubated with MTX and MTX-LDH hybrids respectively over 20 h, resulting in drug accumulation in the G1 phase and certain amount of cell death in the S and G2 phase. However, it is worthy to note that the inhibition of DNA synthesis was more successful in cells treated with MTX-LDH hybrids than those with MTX only, giving 75.09% *vs.* 63.8% at 10 μM/mL, and the gap even increased at higher drug concentrations.

Subsequent study revealed that the MTX-LDH hybrids effective penetration through cell membrane and its high cytotoxicity to cancer cells could be accounted for by not only the cellular retention of the MTX-LDH hybrids in cancer cells, but also the clathrin-mediated endocytosis pathway employed by the hybrids, which is completely different from the cellular uptake mechanism for MTX only. Amazingly, they also found out that the MTX-LDH hybrids could overcome drug resistance [49]. The inhibition of cell proliferation of the MTX resistance cells (HOS/Mtx) treated with free MTX was significant decreased compared to wild-type HOS cells, indicating that drug efficacy was dramatically compromised due to the MTX resistance. On the other hand, both HOS cells and HOS/Mtx cells were remarkably inhibited to a similar degree. These results could also be explained by the uptake mechanism that MTX-LDH hybrids employed. In addition, 5-fluorouracil (5-Fu) was also encapsulated into LDH via co-precipitation in order to further evaluate the potential of drug-LDH complexes as cancer chemotherapy agents [56]. It was showed that both 5-Fu and MTX were intercalated in LDH nanoparticles, achieving higher efficiency of inhibiting cancer cell proliferation in a manner of concentration dependence.

### 4.2. The Delivery of Camptothecin and Podophyllotoxin

Camptothecin (CPT) and Podophyllotoxin (PPT), inhibitors of topoisomerase I and II during DNA synthesis and eventually leading to cell death, have been studied as potential cancer therapeutics [57–62]. Like NADIS, the application of these drugs have been significantly hindered by several deficiencies such as poor water solubility, fast metabolic inactivation, drug resistance and poor bioavailability [62]. However, improvement could be achieved by employing LDH nanoparticles.

Tyner *et al.* have proved that the association with LDH nanoparticles could remarkably enhance the CPT solubility in an aquatic environment [63]. They observed an approximately threefold increase in solubility of CPT-LDH hybrids compared to naked drug, which was further confirmed by Dong *et al.* [64] and Liu *et al.* [65]. Controlled release experiment obtained similar results to NSAIDs associating with LDH, which could be summarized: when drug-LDH complexes expose to a pH 4.2 environment, rapid drug release is obtained due to dissolving of the LDH host; however, sustained drug release could be achieved in a pH 7.2 environment, where the ion exchange was the most responsible for releasing the drugs [63]. Xue *et al.* firstly modified the procedure of PPT-LDHs synthesis in order to achieve higher PPT loading efficiency [66]. They co-precipitated tyrosine (Tyr) with LDH resulting in Tyr incorporation into the interlayer space. Therefore the interlayer space was pre-opened and an environment of inviting drugs was created. Eventually, a drug loading efficiency of 34% *w*/*w* of drug/material was achieved. Preliminary anticancer experiments *in vitro* revealed that tumor cells growth was significantly inhibited by PPT-LDH hybrids, representing higher tumor suppression effects. This theory was further demonstrated by Qin *et al.* [67]. They found out that the PPT-LDH hybrids not only showed higher efficacy to inhibit cancer cells growth compared to naked PPT, but also showed a long-term suppression effect and were more readily internalized into tumor cells *in vitro. In vivo* experiments for evaluating PPT-LDH anti-tumor efficacy were conducted on nude mice bearing HeLa tumor. PPT-LDH hybrids and naked PPT were intraperitoneally injected into mice at a PPT dose of 5 mg/kg body weight. They discovered that remarkable therapeutic tumor suppression 46.39% inhibition rate was achieved by PPT-LDH complexes. In addition, given that high dose of PPT has a life-threatening toxicity to mice [68], it was revealed that the presence of LDH could remarkably reduce the toxicity of PPT, representing the reduction of side effects seen with PPT.

## 5. Recent Applications of LDHS in Medicine

Researchers have used LDHs nanoparticles to impart functionality to a variety of devices in medicine. For example, Li *et al.* have utilized the LDHs as DNA vaccine delivery vector in order to enhance anti-melanoma immune response [69]. Pristine LDHs were prepared by co-precipitation method and were mixed and shaken with pcDNA_3_-OVA plasmid for four hours under 37 °C for DNA/LDH complexes synthesis. Two selected types of mice B16-OVA and C57Bl/6 were separately immunized by the DNA/LDH complex. Seven days after the last immunization, these mice were challenged with B16-OVA melanoma cells. They found out that, after the delivery of the DNA/LDH complexes by the intradermal immunization in two types of mice, the presence of the LDHs could assist the plasmid DNA to induce an enhanced serum antibody response significantly greater than naked DNA vaccine. Over 60% tumor-free and inhibition of melanoma growth were observed in mice that treated with DNA/LDH complexes, whereas no tumor-free mice were found in the control group. They further evaluated this vaccination strategy in a model, which was more analogous to the clinical setting. The therapeutic effect of DNA/LDH complexes immunizations was challenged in mice with pre-established tumor. After a six-day treatment with DNA/LDH complexes in C57BL/6 mice, they observed a significant delay in tumor growth and potent prolongation of mean survival time (from 35 to 50 days). These results suggested that the LDHs mediated cellular transportation could significantly enhance the therapeutic efficacy of DNA immunization, as well as protective immunity against tumor.

Chronic otitis media is a common disease often accompanied by recurrent bacterial infection, resulting in destruction of the middle ear bones. D. Hesse *et al.* employed LDHs as an efficient delivery system for ciprofloxacin in the middle ear *in vivo* [70]. The middle ear implants were individually coated with Mg/Al LDHs loaded with ciprofloxacin. Two groups of Male New Zealand White rabbits (12 each) were infected with *Pseudomonas aeruginosa*, right after the surgery for group 1 and 1 week later after the implantation for group 2. Clinical examination showed that only two rabbits, one of each group, appeared behavioral syndromes and lost 11% of their initial body weight after four days. The clinical neurological examination showed that only one rabbit developed mild imbalance and mild to severe head tilt throughout the whole examination period after infection, whereas three animals showed vestibular signs in group 2 after the infection. Microbiological examination revealed that more animals in group 2 than that in group 1 (yet only 5 *vs.* 1) were detected germ on blood as well as on Gassner agar. Histopathological examination reported that all the 24 animals were accompanied with pulmonary oedema, yet one in group 2 developed severe meningoencephalitis; the remaining rabbits of both groups showed no inflammation of the brain. All these evidences demonstrated that the LDH nanoparticles impregnated with a medical drug can be used as an effective antibiotic delivery system for the challenge of a forced infection *in vivo*. Moreover, it seems that animals underwent concomitant infection during the prostheses implantation developed a better outcome than subjects that were infected with *P. aeruginosa* one week after the implantation, suggesting a fast the LDHs drug release and efficiency.

L. Tammaro *et al.* used LDHs nanoparticles to develop a fluoride-releasing dental material, which could constantly release fluoride over time without any initial toxic burst effect [71]. LDHs loaded with fluoride (LDH-F) were incorporated into commercial light-activated restorative material Bis-GMA/TEGDMA dental resin, Fluoride release study showed that an initial rapid release was observed and followed by a release linearly depending on the time (days). Interestingly, the fluoride release inversely depended on concentration, which could reflect the strong influence of the LDHs lamellar clays morphology. Amazingly, these authors also pointed out that the extrapolated release could last up to one year, with a concentration released every day, which is far from the possible adverse fluoride effect. It has been proposed that the failure of dental restorations is depend on the degree to which the human dental pulp stem cells (hDPSCs) can survive as well as the sensitivity of these cells to injury to trigger an appropriate repair response [72,73]. Given that even a low amount of fluoride can benefit the growth and differentiation of the hDPSCs, authors further investigated whether the active fluoride-releasing materials could affect the proliferation of hDPSC. It was demonstrated that the materials with four different LDH-F concentrations (mass fraction 0.7%, 5%, 10%, and 20%) showed no significant inhibition of proliferation on hDPSC. Moreover, the activity of alkaline phosphatase activity (ALP), an early marker of odontogenic differentiation of pulp cells, gradually increased for 28 days during the growth of cells that had been cultured on fluoride-releasing restorative materials (0.7% and 10%), suggesting that the slow release of small amount of fluoride from active material could positively modulate hDPSCs differentiation.

## 6. Conclusions and Future Directions

LDHs have been widely employed in medical research as drug carriers. In addition, many bio-molecules have been either attached to the surface of or intercalated into LDHs through co-precipitation or anion-exchange reaction. Some molecules that have been used include amino acid and peptides, ATPs, vitamins, and even polysaccharides [4]. Taken advantage of the unique properties of LDH materials, LDH nanoparticles successfully achieve drug delivery into targeted cells *in vitro* and in some case *in vivo* without side-effects [74]. The LDHs based drug delivery systems are therefore well positioned to overcome many hurdles that usually impede successfully drug delivery [4].

Nevertheless, despite numerous advantages, LDH have been encountering obstacles caused by its nature, some of these hurdles critically limit their future application in medicine and other aspects [2,4,37]. Firstly, current studies of drug delivery mediated by LDHs are still focused on intercalating small biomolecules (chemo-drugs, small DNAs and siRNAs and peptides). Only a few successful attempts at large molecules have been reported. This might be due to its restricted inner layer space and limited surface area. Large molecules are believed to attach to the surface of LDH other than being intercalated into inner layers. Therefore, increasing the surface area of LDH could be crucial for loading and delivering large biomolecules. Efforts have been made to modify the LDH synthesis procedure to create LDH nanoparticles with new 3-D structure, the nano-sheet, which is reported to possess larger surface area [75]. However, this is still a field where new research is required. Additionally, since the relatively large plasmids are attached to the LDH surface, they may not be protected from unexpected degradation unless the hybrids are coated with other polymer films to preserve the drug and LDH host being degraded, which is one of the drawbacks of the naked LDHs delivery system. Other drawbacks, including quick aggregation in cultured solution or PBS, easily decomposed in critical environment, rapid drug release after transfection *in vitro*, relatively low targeting transportation, have been greatly limiting the biological applications as well. Even though the surface of the LDH is believed hard to be functionalized due to its simple composition, attempts were made in order to improve LDHs drug delivery efficiency. Tyner *et al.* coated the LDH with disuccinimidyl carbonate (DSC) for improving LDH transportation activity [63]. It was proved that the surface modified samples showed 30-fold more activity compared to control group. Meanwhile, this enhancement to the delivery scheme caused by LDH surface modification could provide potential site-directing of the nano-bybrids. Efforts were also made to slow down the rapid drug release *in vitro* in order to achieve sustained drug liberation and prolong drug efficacy. Alcantara *et al.* encapsulate LDHs by two biopolymers: (i) zein, a highly hydrophobic protein and (ii) alginate, a polysaccharide widely applied for wrapping drugs. Preliminary studies showed that the biocomposite could successfully survive in stomach-like environment (pH 1~2). The hydrophobic nature of zein could prevent water molecules entering the complexes which could cause LDH host decomposition from swelling and eventually controlled drug liberation was achieved [76]. E. Valarezo *et al.* employed poly (ɛ-caprolactone) to coat LDHs via the electrospinning technique [77]. The release curves appeared in a liner manner in the second step even though it was companied with an initial rapid drug release, indicating that the presence of the poly (ɛ-caprolactone) could, to certain extend, compromise the drug release rate. Bao *et al.* synthesized novel LDH/silica core-shell nanostructures (LDH@mSiO_2_), which contained MgAl-LDH nanoplate core and ordered mesoporous silica shell via surfactant-templating method [78]. Studies showed that released drug amount achieved 80% over 10 h after the drug-loaded LDH@mSiO_2_ were placed in PBS buffer (pH = 7.4), whereas the control group liberate 80% of drugs within one hour. Recently, more biocompatible compounds have been employed to functionalize the surface of the LDHs for its medical applications. For instance, the polysaccharide family was attracting a growing attention for LDHs modification to achieve sustainable development. Given the resemblance of their structure with many body components, most polysaccharides are intrinsically biocompatible [79]. Therefore, the common use of the polysaccharides has been justified as binders, fillers and thickeners in solid and liquid formulations and as components for site-specific oral delivery systems [80]. Wicklein *et al.* developed and evaluated lipid-based LDH carriers of efficacious vaccines against influenza A [81]. LDHs were coated with xanthan gum polysaccharide with additional quality of feasible surface modification with biomimetic lipid membrane. Immunogenicity tests in mice revealed that virus immobilized on the lipid bio-hybrid elicited high titers of serum virus-specific antibodies, indicating that a strong immunoreaction was induced. Moreover, Huang *et al.* used liposomes to encapsulate the Dextran-magnetic layered double hydroxide-fluorouracil (DMF), a dextran coated LDHs nanoparticles loaded with the anticancer drug fluorouracil, which concerned the entrapment efficiency and slow-released effect [82]; Ribeiro *et al.* also coated LDHs with pectin for controlled release in the treatment of colon diseases [83]. All these attempts have opened alternatives for the delivery of the drug in the desired location.

Another direction for LDH future development could be achieving “drug co-delivery”. This is a new concept that has been proposed recently for biocompatible inorganic material-based nano-systems in their application of cancer therapies. It is believed that the drug co-delivery method could overcome multidrug resistance (MDR), one of the four severe issues that are encountered during cancer treatment, by concurrently inhibiting the action or reduce the expression of anti-cancer drug efflux transporters and enhancing the activity of the drugs afterwards [84]. Both organic and inorganic material-based co-delivery nano-systems to overcome MDR have been reviewed by several outstanding papers [84–88]. However, few reports have been published about the application of LDH in drug co-delivery due to the related research being still in its infancy. Recently, Li *et al.* firstly developed the co-drug delivery strategy using LDH anion exchange capacity to encapsulate the anti-cancer drug 5-Fu into the inner-layer and load the Allstars Cell Death siRNA (CD-siRNA) onto the surface of LDH nanoparticles [89]. Amazingly, they demonstrated that the combined strategy remarkably enhanced the complexes cytotoxicity to different types of cancer cell lines compared with a single agent. Therefore, the co-delivery of siRNA and anti-cancer drug via LDH has shown great potential as an alternative approach for developing cancer therapy. Furthermore, the development of LDH-mediated drug co-delivery will expand the inorganic drug family and hopefully open a door to achieve an improved organic/inorganic hybrid delivery nano-system.
